# Genetic Polymorphisms of Interleukin-1 Alpha and the Vitamin D Receptor in Mexican Mestizo Patients with Intervertebral Disc Degeneration

**DOI:** 10.1155/2014/302568

**Published:** 2014-11-20

**Authors:** Salvador Cervin Serrano, Dalia González Villareal, Maribel Aguilar-Medina, Jose Guillermo Romero-Navarro, Jose Geovanni Romero Quintana, Eliakym Arámbula Meraz, Ignacio Osuna Ramírez, Veronica Picos-Cárdenas, Julio Granados, Iris Estrada-García, Guzman Sánchez-Schmitz, Rosalío Ramos-Payán

**Affiliations:** ^1^Division of Pain, ISSSTE Hospital, 80200 Culiacán, SIN, Mexico; ^2^Faculty of Biological and Chemical Sciences, Doctoral Programs in Biotechnology and Biomedical Sciences, Autonomous University of Sinaloa, 80010 Culiacán, SIN, Mexico; ^3^Human Genetics Laboratory of the Research Unit, Faculty of Medicine, Autonomous University of Sinaloa, 80019 Culiacán, SIN, Mexico; ^4^Division of Immunogenetics, National Institute of Medical Sciences and Nutrition, 14000 Mexico City, DF, Mexico; ^5^Department of Immunology, National School of Biological Science, National Polytechnic Institute, 11340 Mexico City, DF, Mexico; ^6^Division of Infectious Diseases, Boston Children's Hospital and Harvard Medical School, Harvard University, Boston, MA 02115, USA; ^7^Laboratory of Immunology, Faculty of Biological and Chemical Sciences, Autonomous University of Sinaloa, Avenida de las Américas y Universitarios s/n, Ciudad Universitaria, 80010 Culiacán, SIN, Mexico

## Abstract

Intervertebral disc degeneration (IDD) is the most common diagnosis in patients with back pain, a leading cause of musculoskeletal disability worldwide. Several conditions, such as occupational activities, gender, age, and obesity, have been associated with IDD. However, the development of this disease has strong genetic determinants. In this study, we explore the possible association between rs1800587 (c.-949C>T) of interleukin-1 alpha (*IL1A*) and rs2228570 (c.2T>V) and rs731236 (c.1056T>C) of vitamin D receptor (*VDR*) gene polymorphisms and the development of IDD in northwestern Mexican Mestizo population. Gene polymorphisms were analyzed by polymerase chain reaction followed by restriction fragment length polymorphism, in two groups matched by age and gender: patients with symptomatic lumbar IDD (*n* = 100) and subjects with normal lumbar-spine MRI-scans (*n* = 100). Distribution of the mutated alleles in patients and controls was 27.0% versus 28.0% (*P* = 0.455) for T of rs1800587 (*IL1A*); 53.0% versus 58.0% (*P* = 0.183) for V of rs2228570 (*VDR*); and 18.0% versus 21.0% (*P* = 0.262) for C of rs731236 (*VDR*). Our results showed no association between the studied polymorphisms and IDD in this population. This is the first report on the contribution of gene polymorphisms on IDD in a Mexican population.

## 1. Introduction

Intervertebral disc degeneration (IDD) is one of the most common musculoskeletal disorders. IDD is often diagnosed in patients suffering back pain and is an important contributor to workforce-absence with staggering economic impact worldwide [1, 2].

Spinal degeneration includes both osteoarthritic changes of the facet joint and IDD. Pathophysiology of disc degeneration is not well understood and is thought to occur gradually with aging. Recently, a study in Finnish postmenopausal women showed that higher lumbar bone mineral density (BMD) and *Z*-score from T2-weighted magnetic resonance imaging (MRI) were associated with more severe lumbar disc degeneration at L1–L4 levels [3], and experimental animal models have found that osteoporosis could have a role in IDD progression [4].

Decreased production of extracellular matrix (ECM), increased production of degrading enzymes, and increased expression of inflammatory cytokines in the intervertebral disc are usually observed in IDD. These findings may lead to several alterations including reduced strength of the* nucleus pulposus* and* annulus fibrosus*, dehydration, height reduction, trabecular fissures, end-plate changes, Schmorl nodes, osteophyte formation, spinal stenosis, disc bulging, herniation, and discogenic pain [5, 6]. However, IDD often occurs without associated symptoms as demonstrated by the high incidence of degenerative changes in the asymptomatic population [7–9].

Environmental, behavioral, and anthropometric factors such as gender, obesity, height, occupational activities, and smoking have been associated with a higher risk for developing IDD. In contrast to the relatively minor contribution of these risk factors [10–12], twin-pair studies have found a strong familial aggregation and heritability for IDD [13–16]. In fact, genetic factors account for up to 75% of individual susceptibility to IDD [12, 16–19].

Association studies of genes encoding for structural and functional components of intervertebral disc have highlighted the participation of polymorphisms in IDD [18, 20–22], including collagens I [23], IX [24], and XI [25], aggrecan [26], cartilage intermediate layer protein (CILP) [27], ECM-degrading enzymes such as MMP-3 [28] and MMP9, thrombospondin-2 (THBS2) [29], inflammatory cytokines interleukin-1 alpha (IL-1*α*) [30], IL-18 [21], IL-6, and tumor necrosis factor alpha (TNF-*α*) [31], and vitamin D receptor (VDR) [32].

The hormonal form of vitamin D (calcitriol) plays a key role in mineralization of bone, absorption of calcium from the gut, control of calcium and phosphate homeostasis, and regulation of parathyroid hormone secretion and may affect intervertebral disc maintenance by altering the sulfation of glycosaminoglycans as well as other changes relating to the* nucleus pulposus* ECM. Upon activation by vitamin D, VDR forms a heterodimer with the retinoid-X receptor and binds to hormone response elements on the genome, resulting in the expression of several genes involved in mineral metabolism and other metabolic and immune pathways. Therefore, it seems possible that alterations in the function of the VDR could participate in the pathogenesis of IDD or other diseases where vitamin D plays a role on bone and cartilage maintenance. In fact, several studies have demonstrated a strong association between polymorphisms of the VDR gene with osteoporosis [33], osteoarthritis, and even IDD [32, 34]. Allelic variants of* Fok*I (c.2T>V) of VDR code for structurally different receptors; the product of V allele (mutated) shows relatively increased function, compared with the T allele (larger product). This functional SNP is not in linkage disequilibrium with adjacent polymorphisms and therefore could be considered independent and directly associated with pathological findings [35].

IL-1*α* is a proinflammatory cytokine involved in the regulation of immune responses, inflammatory processes, hematopoiesis, and induction of apoptosis in response to cell injury. IL-1*α*, produced mainly by activated macrophages, neutrophils, and epithelial and endothelial cells, is thought to be involved in the pathogenesis of disc degeneration by increasing the production of ECM degradation enzymes and by inhibiting ECM synthesis [36–38]. Some polymorphisms of the IL-1*α* gene (*IL1A*) have been associated with disc degeneration [30, 39].

The contribution of genetic factors on IDD has been studied mainly in Finnish, Spanish, Chinese, and Japanese populations, but not in Mexico. Therefore, in this work, we selected polymorphisms rs1800587 (c.-949C>T) of* IL1A* and rs2228570 (c.2T>V) and rs731236 (c.1056T>C) of* VDR* to evaluate their potential association with lumbar IDD in a Mestizo population from Sinaloa, a northwestern state from México.

## 2. Materials and Methods

### 2.1. Study Subjects

The population studied consisted of two groups matched by age, gender, and ethnicity: consecutive unrelated patients with symptomatic lumbar IDD (*n* = 100) and nonrelated control individuals without IDD (*n* = 100).

Patients were subjected to T2-weighted MRI of the lumbar spine (L1–L5) to assess disc degeneration by decreased signal intensity, height reduction, bulging, annular tears, herniation, and osteophytes. Patients were diagnosed at the Division of Pain, ISSSTE Hospital, over a period of four years and were classified according to Modic changes on MRI, and the American Society of Neuroradiology (ASNR) criteria [5, 40]. Controls were subjects who attended other divisions of the same hospital, with normal lumbar-spine MRI-scans and lifetime history of no chronic low back pain, accidental back injuries, or spine pathology. For both groups, cases, and controls, the inclusion criteria for age were from 18 to 50 years, both male and female, weight range from 45 to 90 Kg and natives of the northwestern state of Sinaloa, México (for two previous generations). Exclusion criteria were the presence or history of spondylopathologies, spinal tumors, infections, traumatisms, diabetes, systemic lupus erythematosus, rheumatoid arthritis, osteoporosis and other bone diseases, fibromyalgia, and collagen pathologies.

Age, gender, weight, height, and body mass index (BMI) were registered. In accordance with the World Health Organization's categories, subjects with BMI ≥ 25 kg/m^2^ were considered overweight and ≥27 as class-I obese. The Ethical and Research Committee of the ISSSTE Hospital approved this study, and written informed consent, according to the Helsinki Declaration, was obtained from all subjects before their enrollment in the study.

### 2.2. Blood Samples

Peripheral blood was collected by a single venipuncture from subjects. All blood samples were drawn in EDTA containing tubes according to guidelines approved by the Internal Review Committee of the ISSSTE and processed within 1 hour.

### 2.3. DNA Amplification and Restriction Fragment Length Polymorphism

Genomic DNA was isolated from whole blood using the salt precipitation method [41]. Gene polymorphisms were analyzed by polymerase chain reaction followed by restriction fragment length polymorphism (PCR-RFLP). Reaction conditions, primers, and restriction fragments are summarized in Table 1.

A region of 193 bp spanning the rs1800587 polymorphic site at the promoter region of* IL1A* gene in chromosome 2q14 (formerly c.-899C>T or c.-949C>T) was amplified and digested with* Nco*I (2 U for 2 h at 37°C) [31]. Allele C (wild) has one restriction site giving two fragments of 174 and 19 bp, while allele T has no recognition site.

PCR product of 265 bp containing the single nucleotide polymorphism (SNP) rs2228570 at the translation initiation codon of exon 2 of* VDR* gene in chromosome 12q13.11 (formerly c.2T>C) was digested with* Fok*I (1 U for 2 h at 37°C) [31]. Allele T (ancestral) has one restriction site giving two fragments of 197 and 68 bp, while allele V (A, C, or G) has no recognition site. In the same way, the 747 bp amplicon spanning the synonymous polymorphism rs731236 in exon 9 of* VDR* gene (formerly c.352T>C or c.1056T>C) [32] was cleaved with* Taq*I (2 U for 2 h at 37°C). Allele T (ancestral) showed two fragments of 496 and 251 bp, while allele C generated an additional restriction site giving three fragments of 294, 251, and 201 bp.

### 2.4. Statistical Analysis

Demographic and clinical variables of patients and controls were presented as mean ± SD and frequencies. Power calculation showed that a significance level of *P* ≤ 0.05, one-tailed directional, would yield a power of 80% with a sample size of 96 individuals per group [42]. Differences in allelic, genotype, and haplotype frequencies were evaluated using Fisher's exact test. SNPs associations were tested under dominant and recessive genetic models, and odds ratios (OR) with 95% confidence intervals (CI) were used as the measure of association between specific alleles and genotypes with IDD and its clinical subtypes [43]. Significant *P* values were corrected with the Bonferroni test for multiple comparisons [44]. Hardy-Weinberg's equilibrium was calculated by *χ* ^2^ test for all genotype combinations of each SNP in patients and controls. PASW v18.0 (SPSS Inc., Chicago, IL, USA) and Arlequin v3.5.1.2 (Swiss National Science Foundation) software packages were used for analysis.

## 3. Results and Discussion

IDD is a complex pathology, and genetic predisposition could impact the physiological maintenance of intervertebral discs or the control of inflammation and pain [15, 19, 22, 45], ultimately leading to the clinical manifestations of IDD. Genetic studies on IDD have been carried out mainly in population of European (Finnish and Spanish) or Asian (Chinese and Japanese) ancestry, but not in Mexican Mestizos, a major population in North America. Our work, carried out in a northwestern Mexican population, represents the first report of genetic polymorphisms and IDD in a population with mixed native Mexican-European ancestry, also known as Mestizos.

Patients with IDD and control subjects with a mean age of 39.22 (±6.88) versus 39.13 (±6.80) years (*P* = 0.918) were included in this study. In both groups, 89.0% of individuals were female and 11.0% were male. BMI data between patients and controls was 25.99 (±4.33) versus 26.23 (±3.72) kg/m^2^ (*P* = 0.673). Both groups fell into the overweight World Health Organization category for BMI. Covariates followed a normal distribution according to the Kolmogorov-Smirnov test, with no statistically significant differences between the groups. Disc herniation was evident in 80.0% of the patients and herniation with intervertebral osteochondrosis in 20.0%. The percentages of cases based on Modic changes of spine MRI were type I (56.0%), type II (34.0%), and type III (10.0%). Clinical symptomatology included 36.0% with sciatica, 22.0% with lumbago, and 42.0% with lumbosciatica.

Our study on the rs1800587 polymorphism of the* IL1A* showed no significant association with IDD (Table 2). The distribution of T allele in patients and controls was 27.0% versus 28.0% (*P* = 0.455; OR = 0.95; 95% CI = 0.61–1.47). Genotype frequencies between the groups (*P* = 0.455) were 4.0% versus 10.0% for TT; 45.0% versus 35.0% for CT; and 51.0% versus 55.0% for CC, respectively. However, other reports have shown an association between the T allele and IDD. A report on the Finnish population showed that T allele carriers have a higher risk (OR = 2.4) of developing disc bulges than those without it [38]. Another study found that T allele was associated (2.5-fold risk) with Modic changes [46, 47]. An analysis of occupational and genetic factors demonstrated that T allele represented a significant risk factor for the disc degeneration phenotype [48]. A study of a Danish population also found an association of this polymorphism with disc degeneration [39]. In contrast, a case-control study of a population of Spain found no association between T allele and symptomatic lumbar disc herniation [49]. The reason for this inconsistent association between the T allele and IDD may be due to ethnic differences in the studied populations, or by different haplotypes in the promoter or enhancer regions. Mexican Mestizo populations have a high degree of genetic heterogeneity [50], carrying Amerindian and a few European and African HLA haplotypes [51]. Moreover, the population studied by us in Mexico has a particular genetic background, since half of the most common haplotypes found in this population have a proposed European origin, most likely from Spanish origin [52]. Not surprisingly, the frequency of the T allele in our population (28.0%) was similar to the one observed in a Spanish population (35.5%) [49] and alike our results was not associated with IDD.

Genotype frequencies observed between patients and controls (*P* = 0.161) for rs2228570 polymorphism of* VDR* were 20.0% versus 32.0% for VV; 65.0% versus 51.0% for TV; and 15.0% versus 17.0% for TT, respectively (Table 2). Frequencies of V allele were 53.0% versus 58.0% (*P* = 0.183; OR = 0.81; 95% CI = 0.55–1.21). In the case of rs731236 allele C of* VDR* (Table 2), the distribution in patients and controls was 18.0% versus 21.0% (*P* = 0.262; OR = 0.82; 95% CI = 0.49–1.35). Genotype frequencies (*P* = 0.262) were 4.0% versus 3.0% for CC; 27.0% versus 35.0% for TC; and 69.0% versus 62.0% for TT, respectively. In accordance with our allelic frequencies, a recent study on the association between* VDR* polymorphisms and BMD in postmenopausal Mexican women, allelic frequencies of 53% for C of* Fok*I and 27% for V of* Taq*I were reported in the control group [53]. Other reports have shown an association between these* VDR* polymorphisms and IDD. The report in a Finnish population that demonstrated for the first time that* VDR* polymorphism was associated with IDD quantitatively assessed signal intensities of thoracic and lumbar discs, finding those to be 12.9% worse in men with the* Taq*I CC genotype and 9.3% in the men with* Fok*I VV genotype [32]. Reports on Japanese [54], Chinese [55], Turkish [56], English [57], and Australian [58] populations also found association between* VDR* polymorphisms and IDD.

Therefore, for all the studied polymorphisms (Table 2) there were no significant differences in the distribution of alleles and genotypes between the groups (*P* > 0.05) under the genetic models tested. The study of haplotypes showed no differences in frequencies between the groups for any combination of rs2228570 and rs731236 SNPs of* VDR*. The stratified analysis of the data (patients by pathology, symptomatology, and Modic changes) also showed no significant differences (all *P* ≥ 0.05). The genotypes in the groups were not significantly different from the expected distribution for a population in a Hardy-Weinberg equilibrium, with the exception of rs2228570 (*VDR*) in patients (*P* = 0.010).

Our work assessed for the first time the potential contribution of gene polymorphisms on IDD in a Mexican Mestizo population with homogeneous genetic background, since both patients and controls were ethnically and geographically matched. The results showed that no association exists between the studied polymorphisms and IDD in this population. Further analysis of other relevant polymorphisms and more Mestizo populations may contribute to finding the genetic determinants of this disease in our country.

## 4. Conclusion

In conclusion, this is the first report on the potential contribution of gene polymorphisms on IDD in a Mestizo population in Mexico. Our results showed that there is no association of the* IL1A* (rs1800587) and* VDR* (rs2228570 and rs731236) polymorphisms with IDD. The study of more Mestizo populations and more candidate genes may provide further insight into the etiology of the disease.

## Figures and Tables

**Table 1:** Conditions and products of PCR-RFLP.

Gene	SNPs	Primers	Tm	RE	bp	Alleles
			(°C)			(bp)
*IL1A *	rs1800587	5′-GCATGCCATCACACCTAGTT-3′	56	*Nco*I	193	C: 174 and 19
		5′-TTACATATGAGCCTTCCATG-3′				T: 193

*VDR *	rs2228570	5′-AGCTGGCCCTGGCACTGCTCTGCTCT-3′	60	*Fok*I	265	f: 197 and 68
		5′-ATGGAAACACCTTGCTTCTTCTCCCTC-3′				F: 265
	rs731236	5′-CAGAGCATGGACAGGGAGCAAG-3′	59	*Ta* *q* ^*α*^I	746	T: 495 and 251
		5′-GCAACTCCTCATGGCTGAGGTCTCA-3′				t: 294, 251, and 201

Tm: annealing temperature; RE: restriction enzyme.

**Table 2:** Allelic (*af*) and genotype (*gf*) frequencies of *IL1A* and *VDR* polymorphisms in controls subjects and patients with IDD.

SNPs	Patients		Controls	
	(*n* = 100)		(*n* = 100)	
rs1800587 (*IL1A) *				

Alleles	*n*	af	*n*	af

T	53	0.27	55	0.28
C	147	0.74	145	0.73

Genotypes	*n*	gf	*n*	gf

TT	4	0.04	10	0.10
CT	45	0.45	35	0.35
CC	51	0.51	55	0.55

rs2228570 (*VDR*)				

Alleles	*n*	af	*n*	af

V	105	0.53	115	0.58
T	95	0.48	85	0.43

Genotypes	*n*	gf	*n*	gf

VV	20	0.20	32	0.32
TV	65	0.65	51	0.51
TT	15	0.15	17	0.17

rs731236 (*VDR*)				

Alleles	*n*	af	*n*	af

C	35	0.18	41	0.21
T	165	0.83	159	0.80

Genotypes	*n*	gf	*n*	gf

CC	4	0.04	3	0.03
TC	27	0.27	35	0.35
TT	69	0.69	62	0.62
